# The facilitation of constructional meaning in the processing of Mandarin innovative *Bei* construction

**DOI:** 10.3389/fpsyg.2023.1128340

**Published:** 2023-02-09

**Authors:** Qiaoyun Liao, Xin Weng, Chao Dong, Yuxin Zhang

**Affiliations:** ^1^Institute of Linguistics, Shanghai International Studies University, Shanghai, China; ^2^School of English Studies, Shanghai International Studies University, Shanghai, China

**Keywords:** Mandarin innovative *Bei* construction, facilitation, constructional meaning, processing, priming

## Abstract

The processing of Mandarin innovative *Bei* construction in the form of “*Bei* + X” is different from that of the traditional *Bei* construction in that the former activates the constructional meaning which is intrinsically negative. This study thus investigates whether the processing of Mandarin innovative *Bei* construction is facilitated by the access of such emergent negative associations in a self-paced reading experiment with a priming paradigm. In this study, participants firstly read lexical primes in three conditions including construction-related phrases (i.e. phrases expressing the negative constructional meaning of the innovative *Bei* construction), component-related phrases (i.e. phrases expressing the partial literal meaning of the innovative *Bei* construction), and unrelated phrases (i.e. phrases expressing unrelated meaning to the innovative *Bei* construction). They then read sentences where the innovative *Bei* construction was fitted into and finally answered questions. Results showed that the lexical primes conveying the constructional meaning of the innovative *Bei* construction significantly shortened participants’ reading time duration as compared with another two priming conditions. To conclude, the processing of Mandarin innovative *Bei* construction is facilitated by the priming of its constructional meaning, which provides some psychological evidence for the construction-based processing of Mandarin innovative *Bei* construction.

## 1. Introduction

Canonical passive construction expresses the force of an action, typically featured by the passive marker “*被* (*bei*)” followed by a transitive verb in Mandarin. However, other words such as nouns, adjective, and intransitive verbs can also follow the passive marker *Bei*, such as “*被和谐* (“be harmonied,” a person’s opinions that are at odds with prevalent views held in society are either silenced or edited), “*被自杀* (“be suicided,” be mistakenly reported to have taken one’s own life)” and “被小三 (“be mistressed,” be rumored to be a mistress of someone).” This innovative usage is proliferating and gaining acceptance in modern Chinese (Chen, 2010; Qiu, 2017; Chen and Hu, 2020). The above novel constructions are named as Mandarin innovative *Bei* construction with the form of “*Bei* + X” where the X can be filled with nouns, adjectives, and intransitive verbs, which is interpreted as “The subject of the construction involuntarily experiences Event X that brings negative or even detrimental consequences” (Chen and Hu, 2020, p. 91).

Since construction is a form-meaning correspondence, including morphemes, idioms, and words which are partially filled lexis and fully general linguistic patterns (Goldberg, 1995, 1996, 2006, 2019), most studies on the Mandarin innovative *Bei* construction focus on its novel properties, as many researchers have demonstrated that this construction is unique in its syntactic and semantic-pragmatic features. For example, the Mandarin innovative *Bei* construction is considered as an indirect passive structure (Yao et al., 2013), a double violation of form and meaning (Shi, 2013), a light verb structure (Huang and Liu, 2014), and a non-autonomous syntactic structure with semantic changes (Ye, 2019). More importantly, studies compare the innovative *Bei* construction with the canonical passive structure in order to examine its properties. Studies reveal that in terms of syntactic structure, the innovative *Bei* construction is more flexible than the canonical passive structure in that the parts of speech of the lexical item “X” following “*Bei*” can be adjectives, nouns, intransitive verbs as well as transitive verbs (Wang, 2011; Chen, 2017; Chen and Hu, 2020). In terms of semantics, the innovative *Bei* construction expresses various meanings including humor (Yao et al., 2013), mocking (Wang, 2019), sarcasm, and criticism (Wang, 2011; Yao and Song, 2012; Wang, 2019). Among all these associations, one of the most significant aspects is its negative, undesirable, and even detrimental meanings of the subject’s participation in the event X, while the canonical passive structure conveys all positive, neutral, and negative meanings (Chen, 2017; Ye, 2019; Chen and Hu, 2020, p. 96). For example, in the example of “*被自杀* (‘be suicided’)” mentioned above, its extra negative association is “be mistakenly reported to.” By referring to Chen and Hu (2020), these negative associations are defined as the “involuntariness” of the subjects when they are experiencing event X, which mainly refers to three types including “be forced into doing,” “be falsely/mistakenly portrayed as,” and “be thought of/treated as” (p. 85). Taken together, these studies indicate that the “abnormal” form of the innovative *Bei* construction is especially related to its extra negative associations, constructing a form-meaning correspondence.

In addition, other researchers further relate the abovementioned negative associations with certain socio-pragmatic aspects reflected in the construction. For example, Chen (2017) considered that this construction was especially appropriate for the Chinese sociocultural context, as it expresses negative feelings toward the society in a euphemistic way. Similarly, Ye (2019) regarded the innovative *Bei* construction as the dissent against a higher authority in the society. More directly, Chen and Hu (2020) claimed that the innovative *Bei* construction is a “parody of the social reality” (p. 83) associated with ridiculousness, which demonstrates an intrinsic relationship between the negative aspects of this construction and society. Furthermore, what intrinsically relates to this sociality of the innovative *Bei* construction is people’s embodied construal of the society and their interpretation of daily life (Wang, 2011). That is to say, similar to the generation of other constructions such as the causative construction (Liu, 2019) and fictive motion construction (Zhang and Zhang, 2020), the “*Bei* + X” construction also has its embodied base rooted in reality. Specifically, as for the “*Bei* + X” construction, based on the social events (i.e., Event “X”) that are often ridiculous and absurd, it emerges and reflects people’s observation, experience, and cognitive operation of the real world. From this perspective, it can be inferred that Mandarin innovative *Bei* construction has its embodied social ground from which the construction gains its emergent negative meanings.

Finally, some researchers go deeper into the cognitive mechanism of the innovative *Bei* construction. Generally, scholars have taken different perspectives, including the Conceptual Integration Theory (Chen, 2010; Yuan and Liang, 2016), Meme Theory (Chen, 2017), Philosophy of Mind (Qiu, 2017), and generative grammar (Han and Han, 2019), to explain how the innovative *Bei* construction is generated and comprehended. Among these perspectives, here we intend to put a special focus on Construction Grammar (CxG; Goldberg, 1995, 1996, 2006, 2019), whose key assumption is that the meaning of construction cannot be simply inferred from its components, to explore the novelty of the innovative *Bei* construction. It is because this proposal accords with the essential syntactic and semantic feature of the innovative *Bei* construction as a potential form-meaning pairing as discussed before. From this viewpoint, researchers have explored the innovative *Bei* construction by assuming that the cognitive operations underlying the comprehension of this construction are construction coercion (Wang, 2011) and/or reverse construction coercion (Chen and Hu, 2020). Specifically, it is proposed that the “*Bei* + X” structure is coerced into an entirety and should be treated and processed by regarding “*Bei* + X” as a whole instead of two separate components, which hence offers a perspective to explain how the negative associations of this construction emerge.

Taken together, previous studies have provided much interpretation of the novel syntactic-semantic features, social aspects, as well as cognitive mechanisms of Mandarin innovative *Bei* construction, especially focusing on its negative associations. Yet with regard to the exact cognitive processing of this construction, though some studies have tried to clarify that the meaning accessing process of the innovative *Bei* construction is unique from different perspectives, they have not explained whether and how people actually derive the meaning of the innovative *Bei* construction. Given this map and based on the syntactic-semantic novelty of the innovative *Bei* construction, here this study considers CxG as an appropriate perspective to explore the processing of this construction.

According to Goldberg (1995, 1996, 2006, 2019), construction is a form-meaning correspondence. When interpreting constructions, its meaning cannot be strictly predicted from its components (Goldberg, 1996) as the direct combination of meaning components might be unacceptable. For instance, in example (1) (adapted from Chen and Hu, 2020), the Chinese character “*被* (*bei*)” is a marker of passive voice, which should be followed by a transitive verb as required by prescriptive rules, while the following word “*旅游* (travel)” is an intransitive verb in Chinese. Hence the combination of “*被* (*bei*)” and “*旅游* (travel)” is ungrammatical according to the syntactic rules of canonical passive structure in Chinese.

(1) *他被旅游了。.*

“He was traveled (was forced to travel away from home or was mistakenly reported to be traveling).”

However, this contradiction is reconciled by the construction coercion (Goldberg, 1995; Michaelis, 2004) of “*被* (*bei*)” which coerces “*旅游* (travel)” into behaving like a transitive verb and imposes itself onto other parts of the sentence (Wang, 2011; Chen and Hu, 2020). The context then helps to shade the meaning of this construction by defining “*bei*” as “be forced to travel away or be mistakenly reported to be traveling.” In this way, even when the “X” slot (i.e., “travel”) of this construction is a neutral word, there generate extra negative associations of “be forced to” or “be mistakenly reported to.” In other words, this emergent and typical negative meaning possessed by Mandarin innovative *Bei* construction cannot be derived by simply regarding “*被* (*bei*)” and “*旅游* (travel)” separately, as in this case, the coercion process could not occur. Only by regarding “*被旅游* (‘be traveled’)” as a whole can the negative associations be generated and accessed. In this way, it is reasonable to infer that the final interpretation of the innovative *Bei* construction is achieved based on a holistic processing of the “*Bei* + X” combination instead of the independent meanings of “*Bei*” or “X,” which accords with the kernel assumptions of Construction Grammar.

Given the reasonability of investigating the innovative *Bei* construction from the perspective of Construction Grammar, though there is no direct empirical evidence with regard to the processing of Mandarin innovative *Bei* construction, there are some experiments demonstrating the role of the holistic processing of other constructions. For example, Bencini and Goldberg (2000) used a sorting paradigm to investigate whether readers comprehended a sentence’s meaning *via* constructions. Participants were required to sort 16 types of sentences according to their understanding of the overall meanings of the sentences. These sentences varied in their verbs (i.e., throw, slice, get, take) and constructions (i.e., di-transitive, caused-motion, resultative, transitive). Results showed that native speakers favored constructions over verbs as the major classification criteria during the experiment. That is to say, constructions are better predictors of overall sentence meaning than simple verbs.

More importantly, some studies provided evidence more directly for the existence of constructions or constructional meanings. For example, Kaschak and Glenberg (2000)‘s first two experiments explored the processing of “denominal verb” construction (e.g., “crutch”). In pairs of di-transitive and transitive sentences containing those denominal verbs followed by a statement of inference (transfer inference or act-on inference), participants were required to choose one sentence that fit more with the statement (Experiment One) or finish a sentence-paraphrase task and a verb-definition task (Experiment Two). Results of Experiment One revealed that when the inference implied transfer, participants were more likely to choose di-transitive sentences, indicating that the meaning of the construction is not purely tied to the semantics of the verb components, showing that constructions are more likely to be regarded as a whole. Results of Experiment Two showed that participants manifested sensitivity to the holistic constructional meaning in their paraphrases and such constructional meaning shaped the definition imposed on the innovative verbs. These two experiments provide complementary evidence for the existence of constructions and the access of constructional meaning in the comprehension of “denominal verb” construction.

Furthermore, several studies also used the priming paradigm to investigate the processing of constructional meaning. For instance, Eddington and Ruiz de Mendoza (2010) investigated the priming effects of two conditions (same-constructional vs. similar-syntactical) when participants were required to judge whether the argument constructions were correct or not. This experiment showed that when the primes had the same-constructional template with the target, as compared with primes having only similar syntactic structures, participants spent significantly shorter reaction times on the judgment task, which provides evidence for the existence of construction as the psychological entities in language processing. Also, in order to explore how the constructional meaning is accessed, Johnson and Goldberg (2013) used a lexical decision task that created target words with regard to some preceding abstract skeletal constructions (e.g., *He daxed her the norp*), including transitive, di-transitive, caused-motion, and resultative constructions. Three types of target words were created, including high frequency associated words (e.g., Gave), low frequency associated words (e.g., Handed) and semantically related non-associated words (e.g., Transferred) with regard to the construction, resulting in either congruent (e.g., when the skeletal construction and the target word were both di-transitive) or incongruent (e.g., when the skeletal construction was resultative structure but the target word was di-transitive) priming conditions. Results showed significant priming effects for congruent over incongruent target words, both for the associated words with low and high frequency, and even for those semantically related words. The findings lead support for the idea that constructions, as an entirety, convey constructional meanings that are accessed quickly and automatically without explicit instruction. More straightforwardly, in order to investigate the interaction of lexical and constructional meaning in valency coercion processing, Busso et al. (2021) used three types of Italian targets (i.e., construction-associated verbs, lexical-associated verbs, and unrelated verbs as control) primed by coercion instances of Italian argument structure constructions in a lexical decision task. Results revealed that the construction-associated and lexical-associated targets both lead to shorter reaction duration as compared with unrelated targets, which indicates that both construction- or lexical-related meanings can facilitate construction processing. In addition, participants were significantly faster in recognizing construction-associated verbs than lexical-associated ones, further indicating that constructional meaning seems to be more primary than lexical meaning. Therefore, it provides evidence for the function of constructions as a unity instead of separate components in language processing.

Taken together, these studies have provided evidence for the assumption that constructional meaning is derived by processing the construction as a whole, which more or less supports the hypotheses of Construction Grammar. However, regarding the processing of Mandarin innovative *Bei* construction, whether or not it also involves the access to its constructional meaning by regarding “*Bei +* X” as an entirety still lacks empirical investigation. Therefore, in light of the hypothesis of CxG as a possible theoretical account for the processing of the innovative *Bei* construction, this study, based on previous studies, intends to further examine how it is processed by employing a self-paced reading experiment in a priming paradigm. In particular, based on and through the novelty of the negative associations of the Mandarin innovative *Bei* construction, we aim to address the following questions:

(i) Are constructional meanings, which are reflected in the negative associations of Mandarin innovative *Bei* construction, activated in the processing of the construction?

(ii) If yes, how do the constructional meanings influence the processing procedure?

Specifically, we assume that if the negative associations of the constructions are activated and achieved, significant priming effects can be found as the reading time duration of the innovative *Bei* construction will be shorter in the construction-related condition than the other two conditions. It denotes the case that readers treat the “*Bei +* X” construction as a unity, since these negative associations could only be derived by combing “*Bei +* X” as a whole in the construction coercion process as discussed before.

## 2. Method

### 2.1. Participants

Thirty-nine native Chinese speakers (20 females, average age = 24.3, SD = 2.78) participated in this experiment and received financial rewards for their participation. Participants, who were undergraduate university students recruited at Shanghai International Studies University, all had normal or corrected to normal vision. All were right-handed and had no speech-hearing, neurological, or motor disorders.

### 2.2. Materials

Ninety frequently used Mandarin innovative *Bei* constructions were selected from BLCU Corpus Center^1^, in order to ensure that participants were familiar with these expressions. These constructions all consisted of three Chinese characters in the form of “*Bei* + X” in which the “X” is a two-character Chinese word including nouns, verbs, and adjectives. Then these innovative *Bei* expressions were adapted into contexts with the same sentence pattern that included three short clauses (see Table 1).

**Table 1:** An experimental stimuli sample.

Priming conditions		Context				
		Clause 1		Clause 2	Clause 3	
		Person	The innovative *Bei* construction	Post-*Bei* region	Phrase	Phrase
Construction-related	曲解 misunderstanding	李明 Li Ming	被贪污 is mistakenly indicted on corruption	清正廉洁 while he is actually clean and honest	反而 yet	遭受冤枉 he is treated unjustly
Component-related	贿赂 bribing	李明 Li Ming	被贪污 is mistakenly indicted on corruption	清正廉洁 while he is actually clean and honest	反而 yet	遭受冤枉 he is treated unjustly
Unrelated	生病 being sick	李明 Li Ming	被贪污 is mistakenly indicted on corruption	清正廉洁 while he is actually clean and honest	反而 yet	遭受冤枉 he is treated unjustly

For the first clause, it included a two-character noun and an innovative *Bei* construction, with the former referring to a person such as “Li Ming” who underwent the *Bei* construction, and the latter referring to the “*Bei* + X” construction as the target of this experiment. The second clause (i.e., the post-*Bei* region) was a four-character phrase, conveying information that echoed the context. This position was added to allow for the measurement of possible spill-over effects of processing as the faster or easier processing of the targets may also show in the reading time duration of their following words (Wang et al., 2006; Roberts and Liszka, 2013; Jegerski, 2014). The third clause consisted of a two-character phrase followed by a four-character phrase, which served to complete the context. In this way, all materials had the same sentence length and sentence structure. In each context, the reading time duration of the innovative *Bei* construction and the post-*Bei* region was measured and analyzed.

In addition, three types of primes were created (Priming Condition: construction-related vs. component-related vs. unrelated) with regard to each innovative *Bei* construction based on their semantic relatedness with the emergent negative associations of the innovative *Bei* construction (i.e., “*Bei* + X”) as well as the partial lexical meaning of the construction (i.e., “X”). Specifically, the construction-related primes were highly related to the negative meaning of “*Bei* + X” in order to ensure that the construction-related primes reflected the emergent negative associations of the construction. In turn, the component-related primes were highly related to the “X” in order to ensure that the component-related primes reflected the partial lexical meaning of the componential item “X.” Also, the unrelated primes were of very low relatedness with both “*Bei* + X” and “X,” in order to ensure that they did not provide useful semantic activation of the construction.

A group of 18 native Chinese students from Shanghai International Studies University (nine females, average age = 21.83, SD = 1.51) were recruited to rate these priming words based on their semantic relatedness with either the negative associations of “*Bei* + X” or the lexical meaning of “X” in the context on a seven-point scale (1 = extremely unrelated, 7 = extremely related) as well as the plausibility of the context (1 = extremely not plausible, 7 = extremely plausible). Instructions were provided for each participant at the beginning of the test.

As for the construction-related priming condition, priming words with higher ratings with the negative associations of “*Bei* + X” and lower ratings with “X” were selected, in order to ensure that these priming words reflected the negative associations of the construction instead of the lexical meaning of “X.” As for the component-related priming condition, priming words with higher ratings with “X” and lower ratings with the negative associations of “*Bei* + X” were selected, in order to ensure that these priming words reflected the lexical meaning of “X” instead of the negative associations of the construction. As for the unrelated priming condition, priming words with lower ratings with either the negative associations of “*Bei* + X” or “X” were selected, in order to ensure that these priming words did not relate to the meaning of the construction.

Finally, based on the above criteria as well as a high plausibility of context, a total of 60 sets of priming words together with their contexts were selected as the experimental materials. As for the final 60 sets of materials, analyses showed that Priming Condition and Rating Type interacted significantly [*F*(2,929.18) = 14.768, *p* < 0.001]. Further analyses revealed that the construction-related priming words (*M* = 5.07) were significantly more related to the negative associations of “*Bei* + X” than the component-related [*M* = 4.44; *β* = −0.628, SE = 0.155, t(924) = −4.056, *p* < 0.001] and the unrelated (*M* = 2.68) priming words [*β* = 2.389, SE = 0.155, *t*(924) = 15.433, *p* < 0.001], and that the component-related priming words (*M* = 5.20) were significantly more related to the “X” slot than the construction-related [*M* = 4.68; *β* = 0.522, SE = 0.155, *t*(924) = 3.374, *p* = 0.002] and the unrelated (*M* = 2.60) priming words [*β* = 2.600, SE = 0.155, *t*(924) = 16.797, *p* < 0.001]. Also, these three types of priming words did not differ in their strokes and frequency (*p*s > 0.05), and the average plausibility of the entire context was relatively high (*M* = 5.22, *SD* = 3.91). In addition, at the end of the context in each set, one follow-up balanced yes/no question about the innovative *Bei* construction was created in order to detect whether the participants have concentrated on comprehending the materials. For example, in the example provided in Table 1, the question could be “Does Li Ming take a bribe?” and the answer was “No.”

In addition, a total of 90 fillers in the same sentence pattern with the experimental materials were created. The plausibility of these filler sentences was rated by another group of 20 native Chinese students from Shanghai International Studies University (10 females, average age = 21.6, SD = 1.56); and finally, 60 filler sentences were selected based on their relatively high plausibility (*M* = 6.29, SD = 0.91). For each filler sentence, a two-character priming word was created referring to the details of the context. The follow-up questions for fillers were targeted at different segments of the filler sentences, aiming to ensure that participants read every part of the sentence carefully. For example, for this filler sentence “Zhang Liang/is always late for work/without punching in, /which makes the leader annoyed” with the prime “sign in,” the judging task could be “Did you see ‘sign in’ at first?”

The experimental stimuli were divided into three lists based on a Latin-square design and each list was received randomly by one participant. Thus each participant read 120 sentences with primes including 60 experimental sentences and 60 filler sentences. These materials were pseudo-randomized per list in a way that sentences of the same condition would not appear three times consecutively. For each participant, the same target innovative *Bei* construction could appear once in only one context.

### 2.3. Procedure

The experiment was conducted in a quiet room where participants were comfortably seated, approximately 70 cm away from the screen. All materials were presented visually in black on a white background at the center of the computer screen. After instructions were provided, participants pressed the “space” key to move on to the practice block containing four trails.

Each experimental trial started with a “+” of 500 ms followed by a blank screen of 200 ms. Then the priming word appeared and participants read the priming word in a self-paced way by pressing the “space” key, after which a blank screen of 200 ms appeared. After that, participants pressed the “space” key to read the context sentence including three clauses in a self-paced manner. The first clause was presented in two screens including the person’s name and the innovative *Bei* construction; the second clause was presented as a whole; and the third one was presented in two screens including a noun and a phrase. After reading the context sentence, a blank screen of 200 ms appeared, and then the yes/no probing question was presented and lasted until participants made responses (“J” for Yes, “F” for No). After a delay of 800 ms, the next trial was presented. Breaks were allowed during the experiment.

### 2.4. Data collection and analysis

The reading time duration and the accuracy were recorded in E-prime 2.0. Only correctly answered trials were included and analyzed. Reading time duration and accuracy rates that were more than 2.5 standard deviations above and below each group’s means were replaced by that group’s means. As for the reading time duration of the innovative *Bei* construction and the post-*Bei* region as well as accuracy rates, analyses were carried out in the Linear Mixed Model (LMM) with the lme4 (Bates et al., 2015) and lmerTest (Kuznetsova et al., 2017) packages in R (R Core Team, 2022). Priming Condition (construction-related vs. component-related vs. unrelated) was included as the fixed factor, with subject as the random factor.

## 3. Results

Three participants’ data were excluded due to low accuracy rate (<80%). Thus 36 participants’ data were included in the analyses. Results of the accuracy rates, the reading time duration of the innovative *Bei* construction, and post-*Bei* region were presented as follows.

Participants reached generally high accuracy rates (92.67%), with no significant main effect (*p* > 0.05), showing that they had paid equal attention to each condition.

### 3.1. The innovative *Bei* construction

**Figure 1:**
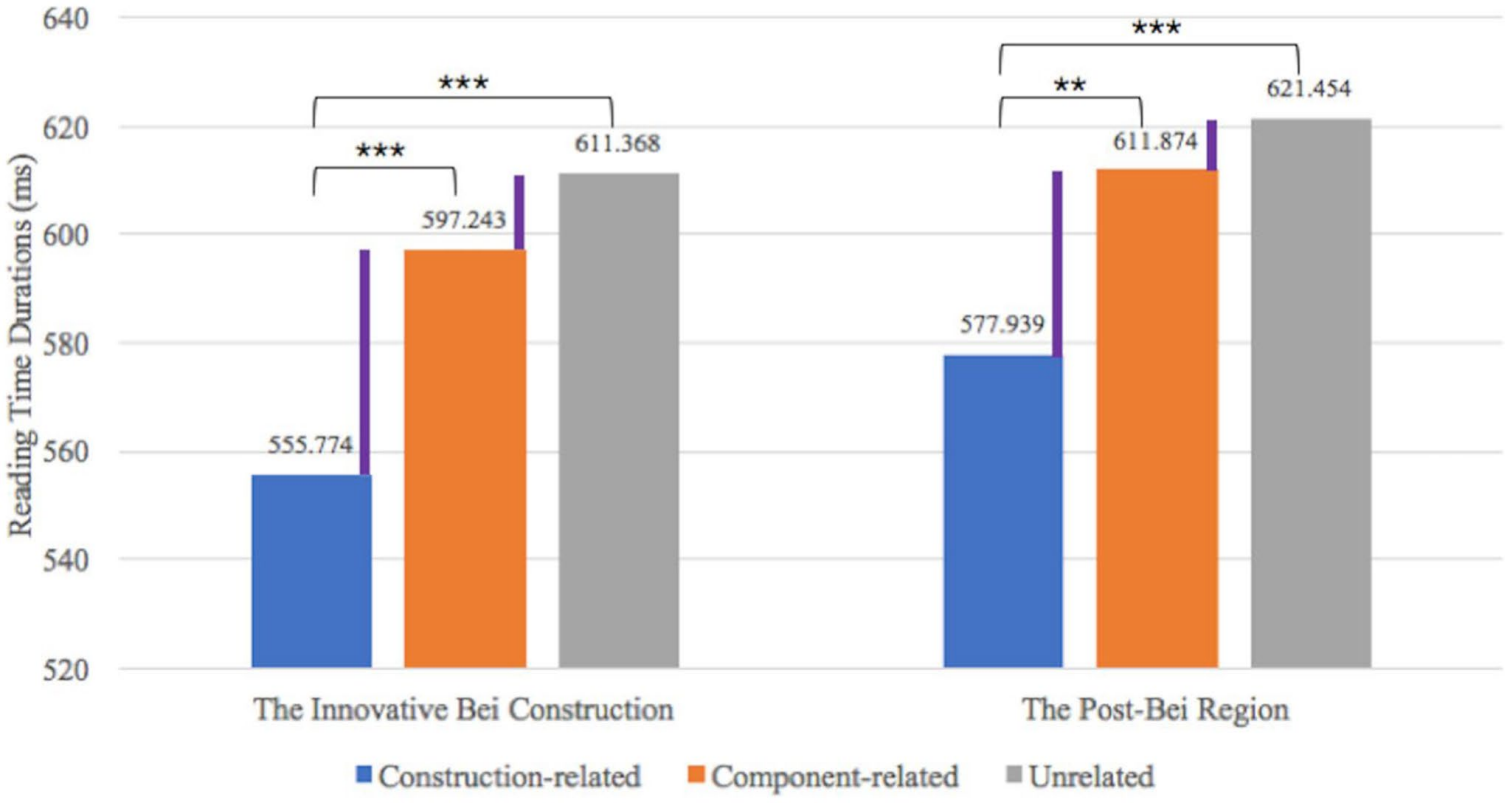
The reading time duration of the innovative *Bei* construction and the post-*Bei* region. The symbols ** and *** refer to the statistical significance of the comparisons. ** refers to the situation when *p* < 0.01; *** refers to the situation when *p* < 0.001.

LMM showed significant effects of Priming Condition [*F*(2,2,124) = 44.537, *p* < 0.001]. Further analyses showed significantly shorter reading time duration for the construction-related condition (*M* = 555.774 ms) than the component-related condition [*M* = 597.243 ms; *β* = 41.5, SE = 6.13, *t*(2126) = 6.769, *p* < 0.001] as well as the unrelated condition [*M* = 611.368 ms; *β* = −55.6, SE = 6.13, *t*(2126) = −9.075, *p* < 0.001]. Besides, the reading time duration for the component-related condition was also slightly shorter than the unrelated condition [*β* = −14.1, SE = 6.13, *t*(2126) = −2.306, *p* = 0.055; see Figure 1].

### 3.2. The post-*Bei* region

Linear Mixed Model also showed a significant effect of Priming Condition [*F*(2,2,124) = 9.528, *p* < 0.001] at the post-*Bei* region. Further analyses also showed significantly shorter reading time duration for the construction-related condition (*M* = 577.939 ms) than the component-related condition [*M* = 611.874 ms; *β* = 33.93, SE = 10.5, *t*(2126) = 3.238, *p* = 0.004] as well as the unrelated condition [*M* = 621.454 ms; *β* = −43.52, SE = 10.5, *t*(2126) = −4.152, *p* < 0.001; see Figure 1].

Taken together, the current experiment revealed significant priming effects of the construction-related primes as contrasted with the unrelated primes both at the reading locations of the innovation *Bei* construction and the post-*Bei* region. In addition, the reading time duration of these two locations was also significantly shorter when primed by construction-related primes than component-related primes.

## 4. Discussion

As hypothesized by Construction Grammer (Goldberg, 1996, 2006), processing Mandarin innovative *Bei* construction involves a holistic storage and processing (Siyanova-Chanturia, 2015) by regarding “*Bei* + X” as a unity. Based on the novelty of this construction that its negative associations are generated by considering this construction as a whole in the construction coercion process, this study explored whether and how such negative associations were activated and achieved in the processing of this construction in a self-paced reading experiment with three types of priming words. Results showed that priming words conveying the negative associations of the innovative *Bei* construction significantly shortened the reading time duration of the “*Bei* + X” construction and the post-*Bei* region than priming words conveying partial componential meaning and unrelated meaning of the construction. These findings indicate that negative associations are activated and achieved in the processing of Mandarin innovative *Bei* construction.

### 4.1. Priming effects of the negative associations of “*Bei* + X” on the processing of innovative *Bei* construction

#### 4.1.1. The facilitation of the negative associations of “*Bei* + X”

As for the target innovative *Bei* construction, analyses revealed a significant priming effect that the negative associations of the construction facilitate participants’ processing of the targeted *Bei* construction as compared with the unrelated condition. Such a priming effect is similar to previous studies of constructions (e.g., Eddington and Ruiz de Mendoza, 2010; Busso et al., 2021) that also report significant priming effects of constructional meaning in the processing of other types of constructions. It indicates that the construction-related primes, which convey the emergent negative associations of the Mandarin innovative *Bei* construction, provide an access to the inference of its constructional meaning before the actual appearance of “*Bei* + X” in the context. It consequently reduces the cognitive efforts required to comprehend the innovative *Bei* construction. Such a facilitative function can be ascribed to the possibility that the nature of the innovative *Bei* construction has more or less been triggered and achieved in advance when participants read the construction-related primes. However, as for the unrelated condition, primes did not provide useful or associative meanings for participants to get access to the constructional meaning of the “*Bei* + X” construction. In this way, in the construction-related condition, when the innovative *Bei* construction appeared again in the context, it is possible and much easier for participants to retrieve the negative associations of the construction from their working memory and apply it to fit in the context where the novel “ungrammatical” *Bei* construction emerges, resulting in shortened processing duration. In addition, the current experiment revealed that this priming effect of the construction-related primes spills over into the reading location of the post-*Bei* region that serves as a buffer to resolve the integration of the innovative *Bei* construction. This downstream effect may further manifest that the facilitative function of the negative associations on the processing of innovative *Bei* construction is relatively stable and consistent.

Furthermore, in the current experiment, the priming effect elicited by the construction-related primes started at the location where participants just read the key “*Bei* + X” construction instead of showing a delayed effect (e.g., starting from the post-*Bei* region). Thus we intend to assume that the activation of the constructional meaning is relatively fast in the processing of Mandarin innovative *Bei* construction. In other words, participants have retrieved and integrated the negative associations into the innovative *Bei* construction before finishing reading the construction itself, further supporting the idea that the accessing of constructional meaning might be quick and autonomous (Johnson and Goldberg, 2013). However, the above inference requires further straightforward experimental evidence and validation.

To conclude, less reading time duration in the construction-related priming condition than the unrelated condition provides evidence that negative associations facilitate the processing of Mandarin innovative *Bei* construction in a relatively instant way.

#### 4.1.2. More contributions of the negative associations of “*Bei* + X” than the lexical meaning of “X”

In the current study, apart from the priming effect of negative associations of “*Bei* + X,” the reading time duration of the “*Bei* + X” region is also shorter when it is primed by the component-related meaning than by the unrelated meaning. Though this is only a marginally significant effect, it still reveals the possibility that partial meaning of the “*Bei* + X” construction (i.e., the lexical meaning of “X”) may also facilitate the processing to some extent. It can be interpreted in terms of the fact that the component-related primes activate part of the meaning of the “*Bei* + X” construction, that is, the meaning of the lexical item “X” as a part of the innovative *Bei* construction (Wang, 2011; Chi and Zhou, 2012). This accords with Busso et al. (2021)‘s results that participants spent less reaction time when the targets were lexically associated to the primes than when the targets were unrelated to the primes.

However, as similarly discovered by Busso et al. (2021), this priming effect of the component-related primes was not so stable or strong as these construction-related primes, because the former was only marginally significant and disappeared at the post-*Bei* region in the current experiment. Also, the reading time duration in the construction-related condition was significantly shorter than that in the component-related condition, further indicating that the emergent negative associations contribute more to the processing of innovative *Bei* construction than the partial lexical meaning of “X.” It is because the activation of the meaning of the lexical item “X” is only an intermediate part in the process of comprehending the innovative *Bei* construction, while the negative associations activated by the construction-related primes represent the final rhetorical meaning of the whole “*Bei* + X” construction. In this way, contrasted to the construction-related primes, component-related primes provide inadequate facilitation to the processing procedure and thus require more cognitive efforts from participants to extract coherent meanings in the comprehension. In this case, the above asymmetry indicates that the processing of the innovative *Bei* construction is influenced by both negative associations of “*Bei* + X” and componential meanings of “X,” but the former seems to be primary as it contributes more to the comprehension, which further emphasizes the importance of negative associations in Mandarin innovative *Bei* construction comprehension.

### 4.2. Evidence for the assumptions of construction grammar

With regard to the processing of constructions, the current study also provides implication for the hypotheses of Construction Grammar as the innovative *Bei* construction display the essential properties of a construction.

Firstly, the innovative *Bei* construction is a unified form-meaning pairing. As mentioned before, the “X” slot in the innovative *Bei* construction can be filled with lexemes of other parts of speech besides the transitive verb, which deviates from the canonical passive structure in Chinese. Hence such a deviation in structure and form occurs together with the emergence of negative associations, constructing a form-meaning correspondence. In the current experiment, the stable facilitation of construction-related primes reveals that participants process “*Bei* + X” as a unified construction. It is because when processing “*Bei* + X,” participants could obtain the negative associations of the construction only by regarding it as a whole. In other words, if participants process “*Bei*” and “X” separately, they could not yield the negative meanings of the construction but only get the lexical meanings of the passive marker and the “X” slot. In a holistic way, these accessed emergent meanings could then be primed by these construction-related primes that convey similar negative associations. Given this map, the current priming effect of the negative associations of “*Bei* + X” provides evidence for the assumption that construction is comprehended as a form-meaning unity.

Secondly, as a construction, the meaning of the innovative *Bei* construction is based on but goes beyond the combination of the meanings of its parts (Goldberg, 1995; Chen and Hu, 2020). Specifically, the current results show that it is the negative associations of the construction instead of the componential lexical meaning that serves as the key through which the innovative *Bei* construction is processed. It indicates that although the piratical lexical meaning of “X” may provide some facilitation to the processing procedure, it does not represent the nature of the construction, for the negative associations of the Mandarin innovative *Bei* construction cannot be strictly predicted from its component parts (Goldberg, 2006). Thus from the aspect of Mandarin innovative *Bei* expressions, this study provides evidence that constructions contain its holistic meaning which cannot be obtained from the components (Goldberg, 2019, pp. 28–42). In addition, this stronger priming effect of construction-related primes than component-related primes may further indicate that the components of the architecture must fit into the designated slots and behave accordingly through coercion (Goldberg, 1995; Chen and Hu, 2020). In the current experiment, the lexical meaning of the component “X” cannot function independently to facilitate construction processing, but is coerced into the passive marker “*Bei*” to produce new meanings (i.e., negative associations). In this way, the combination of “*Bei* + X” can coerce the lexical items “X” into producing systematically related negative meanings, such as “be forced to/be mistakenly reported to,” and can impose constraints on the creation of new constructions (Goldberg, 2006; Wang, 2011). This is also why the component-related primes could only provide limited facilitation to the comprehension of Mandarin innovative *Bei* construction.

Thirdly, as indicated by other studies, the constructional meaning and the componential meaning of constructions may interact with each other through the function of other factors such as “compatibility” (Busso et al., 2021). Though the current study could not address how these two types of meaning indeed interact with each other, the findings of the asymmetric facilitative functions of the negative associations and the componential meanings may at least show that extra negative meanings and partial lexical meanings play different roles in the comprehension of Mandarin innovative *Bei* construction.

### 4.3. The embodied nature of the innovative *Bei* construction

From previous discussions, it is revealed that the processing of the innovative *Bei* construction is facilitated by the negative associations, and thus supports the hypotheses of Construction Grammar. What intrinsically underlies such access and usage of negative associations is an experiential basis grounded in the social reality (Wang, 2011), based on which the cognitive operations of the construction could be carried out and extra meanings could be accessed.

As contrasted with the canonical passive structure that can be interpreted directly, the innovative *Bei* construction is highly reality- and context-based, which requires a clear physical basis. Specifically, the innovative *Bei* construction is interpreted as “The subject of the construction involuntarily experiences Event X” and the “X” denotes an event instead of a state or motion in the canonical passive structure (Chen and Hu, 2020). This event “differs from a verbal process (action or state) in that it is often a specific incident, a real-life story that occurs at a specific time and in a specific place” (Chen and Hu, 2020, p. 92), and is what the subject experiences. For instance, in Example (2) as follows (adapted from Chen and Hu, 2020), the incident is “The registration of a household was outrageously wrong but the local officials refused to correct it.”

(2) *某家人的户口信息错得离谱，当地部门硬是不给改。13岁的女孩于是被长大10岁。.*

“The registration of a household was outrageously wrong but the local officials refused to correct it. Therefore, the 13-year-old girl was grown-up by 10 years (was registered 10 years older than she actually was).”

Through the experience of “X” (e.g., the wrong registration), the subject (e.g., the 13-year-old girl) was mistakenly portrayed as a 23-year-old girl. In this way, the use of the construction “*被长大* (‘be grown-up’)” could be reasonable and understandable. Given this map, we assume that only based on the knowledge that the subject has involuntarily experienced the event denoted by “X,” could the interpretation of the innovative *Bei* construction be successfully achieved. In other words, event “X” is the physical basis of the coherent interpretation of this construction. In addition, these events manifested in the “X” slot are closely related to the social reality from which the innovative construction has emerged. In Example (2), the social reality refers to the dereliction of local officials; and many other innovative *Bei* constructions (e.g., “*被自杀* (‘be suicided’),” “*被和谐* (‘be harmonied’),” etc.) are reflections of the social events occurring at a certain time in China (Chen, 2017; Ye, 2019; Chen and Hu, 2020). This sociality further enhances our assumption that the innovative *Bei* construction requires embodied experience and is grounded in these social realities.

Moreover, based on the experience of event “X,” further cognitive operations are carried out to generate extra negative feelings possessed by the innovative *Bei* construction. Specifically, through a constructional coercion or a reverse constructional coercion (Chen and Hu, 2020), the combination of “*Bei*” and “X” particularly exerts negative impacts and emotions (Wang, 2011; Yao et al., 2013; Huang and Liu, 2014; Chen and Hu, 2020). Specifically, these negative feelings are associated with ridiculousness and absurdity of the social reality, as the subject involved in the event should have avoided such incidents. However, due to many factors, these incidents occur out of control, which subsequently leads to the negative associations of the construction. At the same time, the readers of the construction were “invited to see and experience the specifics of event ‘X’” (Chen and Hu, 2020, p. 103), which further arouses their senses of indignation and helplessness. As revealed in the current experiment, the carrier of these negative associations is the construction-related priming words that convey similar negative feelings aroused by their corresponding innovative *Bei* construction. Hence when reading the construction, participants were virtually experiencing the event “X” and producing negative emotions.

In this way, we regard the processing of the innovative *Bei* construction as an embodied cognitive processing, as it is reality-based and involves participants’ experience of event “X” and their generation of negative emotions.

## 5. Conclusion

This study extends the literature on the comprehension of Mandarin innovative *Bei* constructions by providing psychological evidence of the facilitation of negative associations in its processing. Specifically, priming words conveying the negative associations significantly shortened the reading time duration of the “*Bei* + X” construction and the post-*Bei* region as contrasted with the priming words conveying lexical meanings of “X” and unrelated meanings. These findings indicate that constructional meanings, which are reflected in the negative associations, are activated and achieved to facilitate the processing of Mandarin innovative *Bei* construction in a relatively instant way.

In addition, we should acknowledge that there were several limitations in the current experiment, which may be improved upon in future studies. Regarding this self-paced reading study, it was not able to detect the time course of Mandarin innovative *Bei* construction processing, for example, whether the activation of the constructional meaning is exactly an early and instant process or not. In order to capture more sensitive procedures and provide more fine-grained evidence in Mandarin innovative *Bei* construction processing, eye-tracking and time-locked techniques such as EEG can be used in future studies. In addition, future studies may also improve the experimental materials. Though the materials used in the current study were adapted from corpus and were validated, they were compiled into a fixed sentence pattern for the sake of recording and measuring. Hence future studies should adapt materials of Mandarin innovative *Bei* construction into more natural and daily contexts. Also, it should be noted that other factors that may influence the comprehension of Mandarin innovative *Bei* construction should be included in future studies, such as the degree of familiarity of the construction, the social experiences, and individual differences of participants. Finally, since some of the innovative *Bei* construction may also produce other subtypes of associations such as humor and sarcasm, further studies may also focus on more specific aspects of the emergent associations of this construction.

To conclude, based on current evidence, our data reveal that there is a holistic process in comprehending Mandarin innovative *Bei* construction, as the negative associations of the construction manifest a more stable and strong facilitative function to fit the construction into the contexts. These results provide some new psychological evidence to support the observations of Construction Grammar that the innovative *Bei* construction is a form-meaning pairing whose processing is based on its constructional meaning.

## Data availability statement

The original contributions presented in the study are included in the article/supplementary material, further inquiries can be directed to the corresponding authors.

## Ethics statement

The studies involving human participants were reviewed and approved by the Institute of Linguistics, Shanghai International Studies University. The patients/participants provided their written informed consent to participate in this study.

## Author contributions

CD prepared the materials and collected the data. XW performed all data analyses and wrote the manuscript. All authors contributed to the study conception and design, commented on previous versions of the manuscript, read and approved the final manuscript.

## Funding

This work was supported by the Key Program of the National Social Science Fund of China (grant no. 19AYY011), the Major Scientific Program of Shanghai International Studies University (grant no. 2018114027), and the 5th Tutor Guidance Program of Shanghai International Studies University (grant no. 2022113002).

## Conflict of interest

The authors declare that the research was conducted in the absence of any commercial or financial relationships that could be construed as a potential conflict of interest.

## Publisher’s note

All claims expressed in this article are solely those of the authors and do not necessarily represent those of their affiliated organizations, or those of the publisher, the editors and the reviewers. Any product that may be evaluated in this article, or claim that may be made by its manufacturer, is not guaranteed or endorsed by the publisher.

## Footnotes

^1^ http://bcc.blcu.edu.cn/

## References

[ref1] Bates D. Machler M. Bolker B. M. Walker S. C. (2015). Fitting linear mixed-effects models using lme4. J. Stat. Softw. 67, 1–48. doi: 10.18637/jss.v067.i01

[ref2] Bencini G. M. Goldberg A. E. (2000). The contribution of argument structure constructions to sentence meaning. J. Mem. Lang. 43, 640–651. doi: 10.1006/jmla.2000.2757

[ref3] Busso L. Perek F. Lenci A. (2021). Constructional associations trump lexical associations in processing valency coercion. Cogn. Linguist. 32, 287–318. doi: 10.1515/cog-2020-0050

[ref4] Chen W. B. (2010). A semantic-cognitive investigation of the construction “X is Y”: an exploration of the interface from grammar construction to rhetoric construction. Contemp. Rhetor. 160, 19–26. doi: 10.16027/j.cnki.cn31-2043/h.2012.02.006

[ref5] Chen X. (2017). Extensions of the mandarin passive construction: a memetic account. Pragmatics 2, 59–74. doi: 10.1558/eap.32412

[ref6] Chen R. Hu X. (2020). “Be suicided”: a construction grammar analysis of the innovative bèi construction in mandarin. Cogn. Semant. 6, 83–106. doi: 10.1163/23526416-00502004

[ref7] Chi C. H. Zhou X. J. (2012). The generating mechanism and rhetorical intention of new “bei + X” structure. J. Fujian Norm. Univ. 175, 53–61.

[ref8] Eddington D. Ruiz de Mendoza F. J. (2010). “Argument constructions and language processing: evidence from a priming experiment and pedagogical implications,” in Fostering Language Teaching Efficiency Through Cognitive Linguistics. eds. De Knop S. Boers F. De Rycker A., vol. 17 (Berlin: Mouton de Gruyter), 213.

[ref9] Goldberg A. E. (1995). Constructions: A Construction Grammar Approach to Argument Structure. Chicago, IL: University of Chicago Press.

[ref10] Goldberg A. E. (1996). Words by default: optimizing constraints and the Persian complex predicate. In Annual meeting of the Berkeley linguistics society 22, 132–146). Berkeley: Berkeley Linguistics Society.

[ref11] Goldberg A. E. (2006). Constructions at Work: The Nature of Generalization in Language. Oxford: Oxford University Press.

[ref12] Goldberg A. E. (2019). Explain Me This: Creativity, Competition, and the Partial Productivity of Constructions. Princeton: Princeton University Press.

[ref13] Han Y. Han J. (2019). The syntactic derivation of non-canonical Bei -sentences in mandarin. Foreign Lang. China 16, 39–47. doi: 10.13564/j.cnki.issn.1672-9382.2019.02.007

[ref14] Huang Z. D. Liu N. (2014). The syntax and semantics of the new non-canonical Bei XX construction. Lang. Sci. 70, 225–241.

[ref15] Jegerski J. (2014). “Self-paced reading,” in Research Methods in Second Language Psycholinguistics. eds. Jegerski J. Van Patten B. (New York: Routledge), 36–65.

[ref16] Johnson M. A. Goldberg A. E. (2013). Evidence for automatic accessing of constructional meaning: jabberwocky sentences prime associated verbs. Lang. Cogn. Process. 28, 1439–1452. doi: 10.1080/01690965.2012.717632

[ref17] Kaschak M. P. Glenberg A. M. (2000). Constructing meaning: the role of affordances and grammatical constructions in sentence comprehension. J. Mem. Lang. 43, 508–529. doi: 10.1006/jmla.2000.2705

[ref18] Kuznetsova A. Brockhoff P. B. Christensen R. H. B. (2017). lmerTest package: tests in linear mixed effects models. J. Stat. Softw. 82, 1–26. doi: 10.18637/jss.v082.i13

[ref19] Liu Y. F. (2019). A study of conceptual overlap of causative Jianyu construction from the perspective of embodied-cognitive linguistics. Foreign Lang. Lit. 5, 15–21. doi: 10.3969/j.issn.1674-6414.2019.05.003

[ref20] Michaelis L. A. (2004). Type shifting in construction grammar: an integrated approach to aspectual coercion. Cogn. Linguist. 15, 1–67. doi: 10.1515/cogl.2004.001

[ref21] Qiu J. (2017). Mind-philosophical approach to new mandarin passive construction and its English translation. J. Sichuan Int. Stud. Univ. 33, 90–96. doi: 10.3969/j.issn.1674-6414.2017.05.016

[ref22] R Core Team. (2022). R: A Language and Environment for Statistical Computing. R Foundation for Statistical Computing. Vienna, Austria. Available at: https://www.R-project.org/.

[ref23] Roberts L. Liszka S. (2013). Processing tense/aspect agreement violations online in the second language: a self-paced reading study with French and German L2 learners of English. Second Lang. Res. 29, 413–439. doi: 10.1177/0267658313503171

[ref24] Shi C. H. (2013). The generation mechanism, semantic interpretation and pragmatic effects of the new “Bei” construction. Contemp. Rhetor. 1:12-28. doi: 10.16027/j.cnki.cn31-2043/h.2013.01.003

[ref25] Siyanova-Chanturia A. (2015). On the ‘holistic’ nature of formulaic language. Corpus Linguist. Linguist. Theory 11, 285–301. doi: 10.1515/cllt-2014-0016

[ref26] Wang Y. (2011). Analysis of “new construction with *Bei*” via lexical coercion: a research on “*Bei Ziyuan*” in cognitive construction grammar. J. Foreign Lang. 34, 13–20.

[ref27] Wang Y. G. (2019). “*Bei* X” Snowclones in speech-act/epistemic domains and constructional coercion. J. Foreign Lang. 2, 34–44. doi: 10.3969/j.issn.1004-5139.2019.02.004

[ref28] Wang S. P. Chen H. Z. Yang J. M. Wu Y. Wang R. M. (2006). Immediacy of integration in reading Chinese. Acta Psychol. Sin. 5, 645–653.

[ref29] Yao J. Song J. (2012). The identification and interpretation of ironical Chinese *Bei*-passives. Foreign Lang. Res. 1, 55–58. doi: 10.16263/j.cnki.23-1071/h.2012.01.014

[ref30] Yao J. Song J. Singh M. (2013). The ironical Chinese Bei-construction and its accessibility to English speakers. J. Pragmat. 55, 195–209. doi: 10.1016/j.pragma.2013.06.003

[ref31] Ye Z. D. (2019). The emergence of expressible agency and irony in Today’s China: a semantic explanation of the new Bèi -construction. Aust. J. Linguist. 39, 57–78. doi: 10.1080/07268602.2019.1542933

[ref32] Yuan H. M. Liang J. Y. (2016). An analysis of the conceptual integration of the constructional meaning of “*Bei* + X”. Foreign Lang. Res. 33, 33–39. doi: 10.13978/j.cnki.wyyj.2016.01.026

[ref33] Zhang K. D. Zhang X. (2020). The embodiment-cognition and interactivity of fictive motion construction. Foreign Lang. Educ. 5, 6–10. doi: 10.16362/j.cnki.cn61-1023/h.2020.05.002

